# Histone deacetylase 1 induced by neddylation inhibition contributes to drug resistance in acute myelogenous leukemia

**DOI:** 10.1186/s12964-019-0393-8

**Published:** 2019-07-29

**Authors:** Qiu-yu Lai, Ying-zhi He, Xiong-wen Peng, Xuan Zhou, Dan Liang, Liang Wang

**Affiliations:** 1 Department of Hematology, ZhuJiang Hospital of Southern Medical Univeristy, No. 253 GongyeDadaoZhong, 510280 Guangzhou, Guangdong People’s Republic of China; 2 Department of Hematologic Oncology, Sun Yat-sen University Cancer Center, Guangzhou, China

**Keywords:** Acute myelogenous leukemia, Drug resistance, HDAC1, Neddylation, Ubiquitination

## Abstract

**Objective:**

This study aimed to investigate the function and mechanism of neddylation of HDAC1 underlying drug resistance of AML cells.

**Methods:**

Evaluation experiments of effects of HDAC1 on drug resistance of AML cells were performed with AML cell transfected with constructs overexpressing HDAC1 or multi-drug resistance AML cells transfected with siRNA for HDAC1 through observing cell viability, percentage of apoptotic cell, doxorubicin-releasing index and multidrug resistance associated protein 1 (MRP1) expression. Neddylation or ubiquitination of HDAC1 was determined by immunoprecipitation or Ni2^+^ pull down assay followed by western blot. The role of HDAC1 was in vivo confirmed by xenograft in mice.

**Results:**

HDAC1 was significantly upregulated in refractory AML patients, and in drug-resistant AML cells (HL-60/ADM and K562/A02). Intracellular HDAC1 expression promoted doxorubicin resistance of HL-60, K562, and primary bone marrow cells (BMCs) of remission AML patients as shown by increasing cell viability and doxorubicin-releasing index, inhibiting cell apoptosis. Moreover, HDAC1 protein level in AML cells was regulated by the Nedd8-mediated neddylation and ubiquitination, which further promoted HDAC1 degradation. In vivo, HDAC1 overexpression significantly increased doxorubicin resistance; while HDACs inhibitor Panobinostat markedly improved the inhibitory effect of doxorubicin on tumor growth. Furthermore, HDAC1 silencing by Panobinostat and/or lentivirus mediated RNA interference against HDAC1 effectively reduced doxorubicin resistance, resulting in the inhibition of tumor growth in AML bearing mice.

**Conclusion:**

Our findings suggested that HDAC1 contributed to the multidrug resistance of AML and its function turnover was regulated, at least in part, by post-translational modifications, including neddylation and ubiquitination.

**Electronic supplementary material:**

The online version of this article (10.1186/s12964-019-0393-8) contains supplementary material, which is available to authorized users.

## Background

Acute myelogenous leukemia (AML) is a common cancer, which is frequently accompanied by a poor prognosis among young people and adults [1, 2]. With developments in chemotherapy, remission rates of AML have increased dramatically (53–70%), with the curative effect of treatment dependent on the patient’s age [3]. However, most patients with AML will relapse due to multiple drug resistance with enhancing cancer cell capability to repair DNA damage and resistant to apoptosis [4, 5]. Therefore, studies of the mechanism underlying chemotherapy resistance in AML will provide the potential new therapies for prevention and treatment of AML.

According to the literature, post-translation genetic modification (e.g., methylation, acetylation, and phosphorylation) of chromatin-modifying proteins, including histones and non-histones, plays an important role in the pathogenesis of various diseases [6, 7] as well as in carcinogenesis, including AML [8, 9]. Recent research has focused on the role of histone deacetylases (HDACs), catalytic enzymes involved in deacetylation modification of histone and non-histone substrates, in health and disease, by regulating cellular hemostasis and apoptosis [10]. There are 11 known HDAC enzymes in mammals, named HDAC1–11 [11]. Several patterns of HDAC have been identified in AML cells [12]. Also HDAC-mediated modification of chromatin has been shown to be involved in the occurrence and development of AML [13, 14]. Consequently, various AML treatment strategies have been developed based on different classes of HDAC inhibitors [13, 14]. Except the pivotal role of HDACs in AML indicated by these studies, a recent study provided evidence that the inhibition of HDACs was negatively correlated with AML resistance to chemotherapy [15]. However, the way in which the expression pattern of HDACs contributes to multidrug resistance of AML remains unclear.

The regulation of protein function by HDACs depends on maintaining a cellular balance between protein synthesis and protein degradation. Post-translational modification is the dominant mechanism responsible for the regulation of protein levels and functions, with neddylation and ubiquitination the two most important methods [16]. Neddylation and ubiquitination may exist alone or together in cells [17]. During ubiquitination, proteins targeted for degradation are first labeled by ubiquitin. An ubiquitin-like molecule, Nedd8 (neural precursor cell expressed developmentally down-regulated 8), that targets proteins for degradation has also been identified. The neddylation of HDACs has also been observed in some cells [18, 19]. To date, there is no evidence that HDACs are modified by Nedd8 or ubiquitin in AML cells.

The aim of the present study was to investigate the potential involvement of HDACs in multidrug resistance of AML, in addition to the effect of neddylation and ubiquitination on intracellular HDAC expression and drug sensitivity of AML cells.

## Materials and methods

### Patients and collection of bone marrow samples

Ten AML patients who received treatment in our hospital were included in this study, 5 in remission group (2 female and 3 male) and 5 in refractory group (2 female and 3 male). Patients in refractory group had experienced multiple recurrences. Patients in remission group were not relapsed for 1 year after remission. The median age of AML patients was 55 years (range 19–71 years). All cases were M2 type. All cases were CD44+. There were two cases of frequent genetic mutation in *FLT3* of internal tandem duplications (ITDs) (FLT3-ITD+), and four cases of gene rearrangements in *MLL* (MLL+), averaging distribution between the two groups. Genetic background of all patients was similar.

Bone marrow samples were available both at the diagnosis time prior to treatment and the relapsed/refractory state from AML patients after they signed informed consent forms. The experimental protocols were approved by the ethics committee of ZhuJiang Hospital of Southern Medical University.

### Cell culture and treatment

AML cell lines (HL-60 and K562) and matching multidrug-resistant cell lines (HL-60/ADM and K562/A02) were obtained from the Chinese Academy of Medical Sciences and Peking Union Medical College. All the cells were cultured in RPMI 1640 medium, supplemented with 10% fetal calf serum and incubated at 37 °C, 5% CO_2_ in a humidified incubator. In order to maintain the MDR phenotype, the medium of drug-resistant cell lines were supplementary with doxorubicin 2 weeks before experiments. Bone marrow cells (BMCs) were isolated by density gradient centrifugation from the bone marrow samples of the AML patients (remission or refractory) and cultured in basal RPMI 1640 medium, supplemented with 5% fetal bovine serum. Fresh medium was added every two or three days.

### Western blot

Total protein was isolated from the bone marrow samples of the AML patients or AML cells using RIPA lysis solution, supplemented with protease and phosphatase inhibitors. The protein concentration was established using a BCA kit. Equivalent quantities of protein samples were then subjected to 10–12% SDS-polyacrylamide gel electrophoresis and transferred to nitrocellulose membranes (Millipore). The membranes were then stained with primary antibodies against HDAC1–11 (Abcam), acetyle-histone 3, histone 3, acetyle-histone 4, histone 4, Apaf-1 and MRP1 (Cell Signaling Technology), followed by immune-probed using a horseradish peroxidase-conjugated secondary antibody. β-actin (Abcam) was used as an internal reference gene. The target protein was visualized using an ECL Western blotting substrate kit (Biovision).

### MTT assay

Cell viability was detected by an MTT assay. In brief, the cells were incubated on a 96-well plate until 80% cell confluence and then subjected to transfection and doxorubicin treatment. After incubation, cell viability was analyzed using a Cell Proliferation Kit 1 (MTT) (Sigma-Aldrich). MTT solution was added to each well and cultured for 4 h. The reaction was stopped, and DMSO was added to dissolve formazan crystals. The final mixture was analyzed using an enzyme-linked immunometric meter at 490 nm.

### Flow cytometry analysis of cell apoptosis and the doxorubicin-releasing index

Cell apoptosis were analyzed by flow cytometry using an Annexin V-FITC/PI Apoptosis Kit (BioVision), according to the manufacturer’s protocols. Briefly, cells that have been treated by doxorubicin or HDACs inhibitors or pcDNA-HDAC1 were collected and washed with cold PBS following Apoptosis Kit treatment. Cells were firstly orderly incubated with annexin-V and PI in binding buffer. After staining, cells were analyzed on FacsCalibur flow cytometer (Becton Dickinson).

Primary BMCs from all patients were characterized using monoclonal antibodies (mouse anti-human) CD44 PE (phycoerythrin). Before analyzed on flow cytometer in the immunophenotypic panel, BMCs were incubated with antibody with unstained used as control.

For the analysis of intracellular doxorubicin, the cells were seeded in a six-well plate and subjected to transfection with pcDNA-HDAC1 or si-HDAC1 or treatment with HDAC inhibitors (AR-42 or Panobinostat) or specific inhibitor for HDAC1 before the addition of doxorubicin. After incubation, the cells were harvested, and the fluorescence intensity of doxorubicin was detected by flow cytometry. The florescence intensity of intracellular doxorubicin was presented as the doxorubicin-releasing index, which was calculated according to the formula: the doxorubicin-releasing index = (accumulation value-retention value)/accumulation value.

### Cell transfection

To evaluate the role of HDAC1 in doxorubicin sensitivity of AML cells, pcDNA plasmid constructs over-encoding HDAC1 or siRNA constructs silencing HDAC1 (si-HDAC1) were respectively transfected into AML cells or drug-resistant AML cells before doxorubicin treatment using Lipofectamine® 2000 (Thermo Fisher Scientific). Simultaneous transfection of an empty pcDNA plasmid or si-control into the cells was used as a control. To explore the neddylation and ubiquitination of HDAC1, constructs encoding HDAC1, His-ubiquitin (His-Ub), Flag-NUB1, Myc-Mdm2, and NEDP1 were generated by cloning these genes in a lentiviral-based vector and then transfecting them into HL-60 cells.

### His purification

To verify that HDAC1 was a substrate for Nedd8, HL-60 cells were co-transfected with Flag-HDAC1 and different doses of His-Nedd8. After transfection, the cells were harvested for preparation of lysates. The substrate of His-Nedd8 in lysates was purified using Ni^2+^-NTA agarose beads (Qiagen) and subjected to a Western blot analysis of Nedd8 and HDAC1 expression. Nedd8 antibody was obtained from Abcam. His purification from HL-60 cells transfected with His-Nedd8 was used to detect endogenous HDAC1. To further demonstrate the use of HDAC1 as a substrate for Nedd8, 1 μM or 5 μM MLN4924 (an inhibitor of Nedd8-activating enzyme) was added before Flag-HDAC1 and His-Nedd8 transfection.

### Immunoprecipitation

Following cell lysis by RIPA lysis solution, supplemented with protease and phosphatase inhibitors, proteins were obtained by centrifugation at 4 °C. The protein concentration was established using a BCA kit. Equivalent quantities of protein were incubated with an antibody overnight at 4 °C. The next day, they were incubated with Protein G/A agarose beads on a horizontal rotator. Beads-antibodies compound was harvested and freed out the antigen and antibody for Western blot analysis with antibodies against Nedd8 and HDAC1.

### Mouse tumor xenograft model

A HL-60 xenograft overexpressing HDAC1 or a HL-60/ADM xenograft with silencing of HDAC1 was subcutaneously transplanted into the right flank of six-week-old BALB/c nude mice (six mice of each treatment; Shanghai Laboratory Animal Research Center). Approximate 1 × 10^7^ cells were subcutaneously injected into the right posterior flank of each mouse. The experimental protocols involving animal studies were approved by the ethics committee at ZhuJiang Hospital of Southern Medical University. All animal experiments were performed in accordance with the guidelines of the Care and Use of Laboratory Animals of the National Institute of Health. On day 10 after transplantation, the animals received an intravenous injection of doxorubicin and oral administration of Panobinostat once a day. The tumoral size was measured each week, starting from one week after the injections, and the tumoral volume was calculated. After four weeks, the animals were sacrificed, and the xenograft was removed and imaged.

### Statistical analysis

Data were obtained from at least three independent experiments and are presented as the mean ± standard deviation (SD). The data analysis was performed with SPSS 16.0. Comparisons were analyzed using a Student *t*-test or a one way analysis of variance. A *p*-value less than 0.05 are considered statistically significant.

## Results

### Differential expression of HDACs

**Fig. 1:**
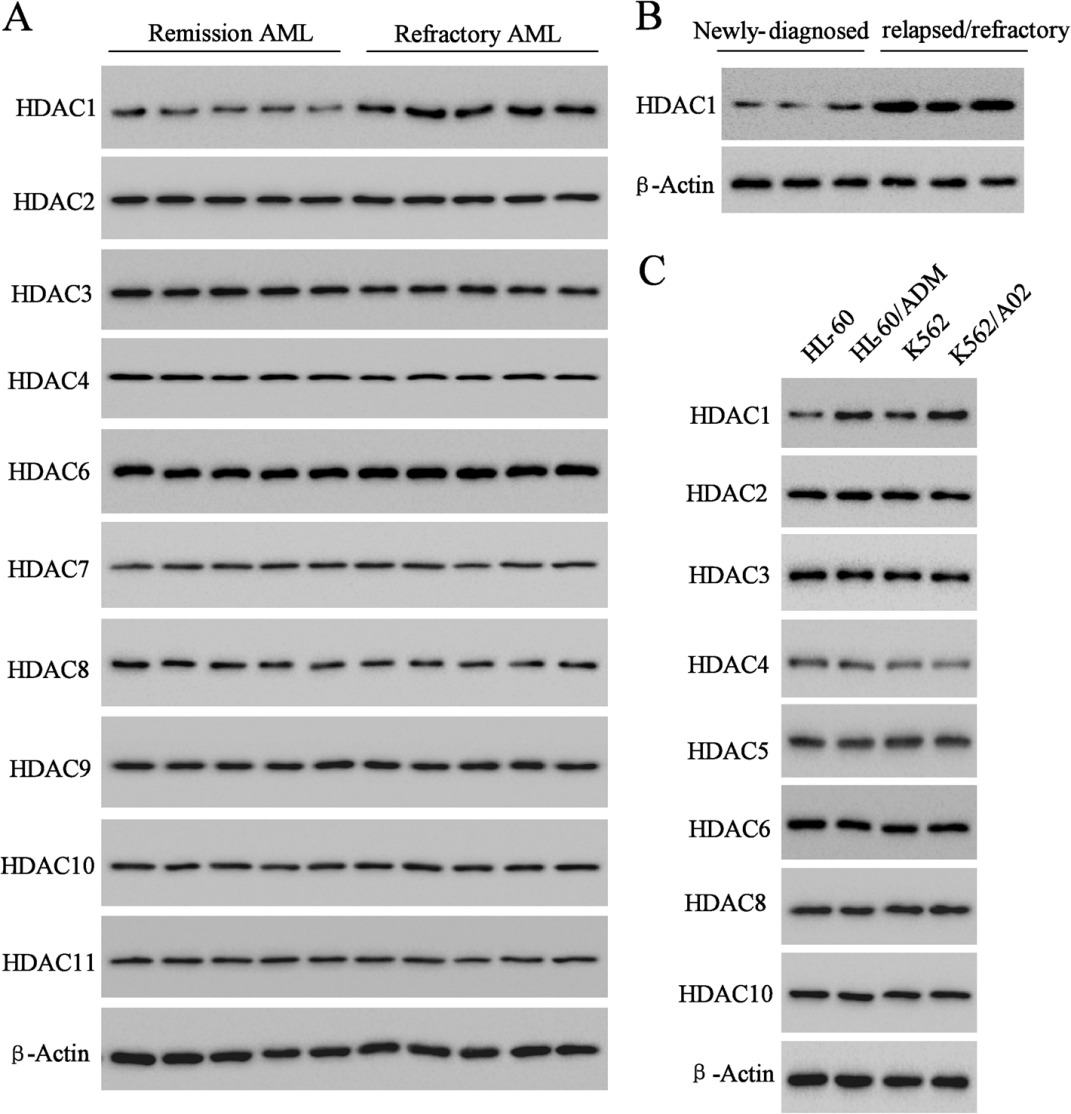
The expression profile of HDACs in AML. Western blot analyzed the different expression level of (**a**) HDACs between remission AML and refractory AML patients, (**b**) HDAC1 between newly-diagnosed and refractory AML and (**c**) HDACs between AML cell lines (HL-60 and K562) and multidrug-resistant AML cell lines (HL-60/ADM and K562/A02)

To determine the potential involvement of HDAC expression patterns in multidrug resistance of AML, the expression of HDACs (HDAC1–11) in five cases of refractory AML was compared to that of five cases of remission AML. As shown in Fig. 1a, HDAC1–4 and HDAC6–11 were widely detected, and HDAC5 was not expressed (data not shown). Among the HDACs that were expressed, HDAC1 had the greatest increase in expression level in refractory AML compared to that in remission AML. HDAC1 in refractory AML was also expressed higher level than that in newly-diagnosed AML (Fig. 1b). Importantly, the higher expression of HDAC1 was also observed in the multidrug-resistant AML cell lines (HL-60/ADM and K562/A02) and nondrug-resistant AML cell lines (HL-60 and K562) (Fig. 1c). HL-60/ADM and K562/A02 resistant to doxorubicin was confirmed by the measurement of cell growth inhibition rate of HL-60, K562, K562/A02 and HL-60/ADM cells in logarithmic growth phase after 48 h treatment with doxorubicin of different concentrations, in which doxorubicin (IC50) of HL-60 /ADM cells (9.39 μg/ml) was 100 times greater than that of HL-60 cells (0.07 μg/ml) and that of K562/A02 cells (25.42 μg/ml) was 100 times greater than that of K562 cells (0.18 μg/ml) Additional file 4: Figure S4. Moreover, in refractory patient samples, expression levels of acetyle-histone 3 (Ac-H3) and acetyle-histone 4 (Ac-H4) were significantly increased, and that of histone 4 (H4), and histone 3 (H3) had no obvious change (Additional file 1: Figure S1A & B).

### HDAC1 overexpression increased doxorubicin resistance of AML cells

**Fig. 2:**
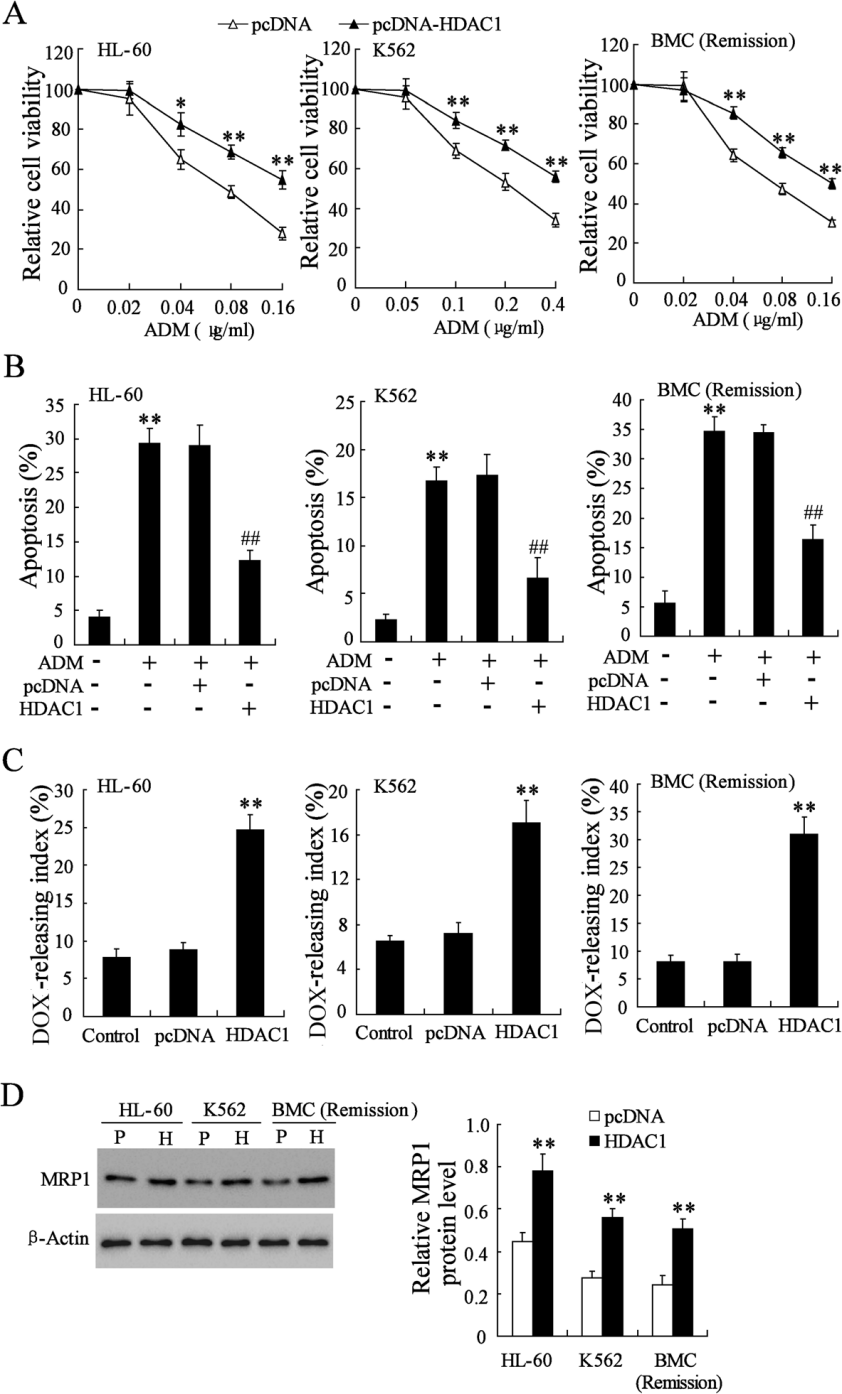
Effect of HDAC1 overexpression on doxorubicin resistance of HL-60, K562, and primary BMCs of remission AML. **a** Relative cell viability of pcDNA-HDAC1 transfected AML cells after different doses of doxorubicin treatment. **b** Percentage of apoptotic cells in pcDNA-HDAC1 transfected AML cells after treatment with 0.1 μg/ml of doxorubicin. **c** doxorubicin-releasing index of pcDNA-HDAC1 transfected AML cells. **d** MRP1 protein expression in pcDNA-HDAC1 transfected AML cells. P, pcDNA; H, pcDNA-HDAC1. DOX, doxorubicin. Data are the mean ± SD of three independent experiments in AML cells. ^*^P < 0.05, ^**^P < 0.01 vs. pcDNA

In the attempt of identify the main role of HDAC1 in the drug resistance of AML, the nondrug-resistant AML cell lines (HL-60 and K562) and primary BMCs of remission AML patients were transfected with pcDNA-HDAC1 to stably express HDAC1 (Additional file 1: Figure S1C). The effects of HDAC1 on the doxorubicin resistance of AML cells were assessed by the cell viability, apoptosis and doxorubicin-releasing index. As shown by the results of MTT assay, the cell viability of HL-60, K562, and primary BMCs were decreased obviously by doxorubicin treatment in a dose-dependent manner; while HDAC1 overexpression significantly rescued the doxorubicin-induced decrease in cell viability (Fig. 2a). The results of flow cytometry showed that HDAC1 overexpression alleviated the pro-apoptotic action of doxorubicin (Fig. 2b) and increased the doxorubicin-releasing index in HL-60, K562, and primary BMCs (Fig. 2c). In addition, multidrug resistance associated protein 1 (MRP1), a gene involved in drug resistance [20] and being found expressed high level in refractory AML (Additional file 1: Figure S1D), was upregulated by HDAC1 overexpression (Fig. 2d). These data suggested that the doxorubicin resistance in nondrug-resistant AML cells was increased by HDAC1 overexpression.

### HDAC1 knockdown decreased doxorubicin resistance of multidrug-resistant AML cells

**Fig. 3:**
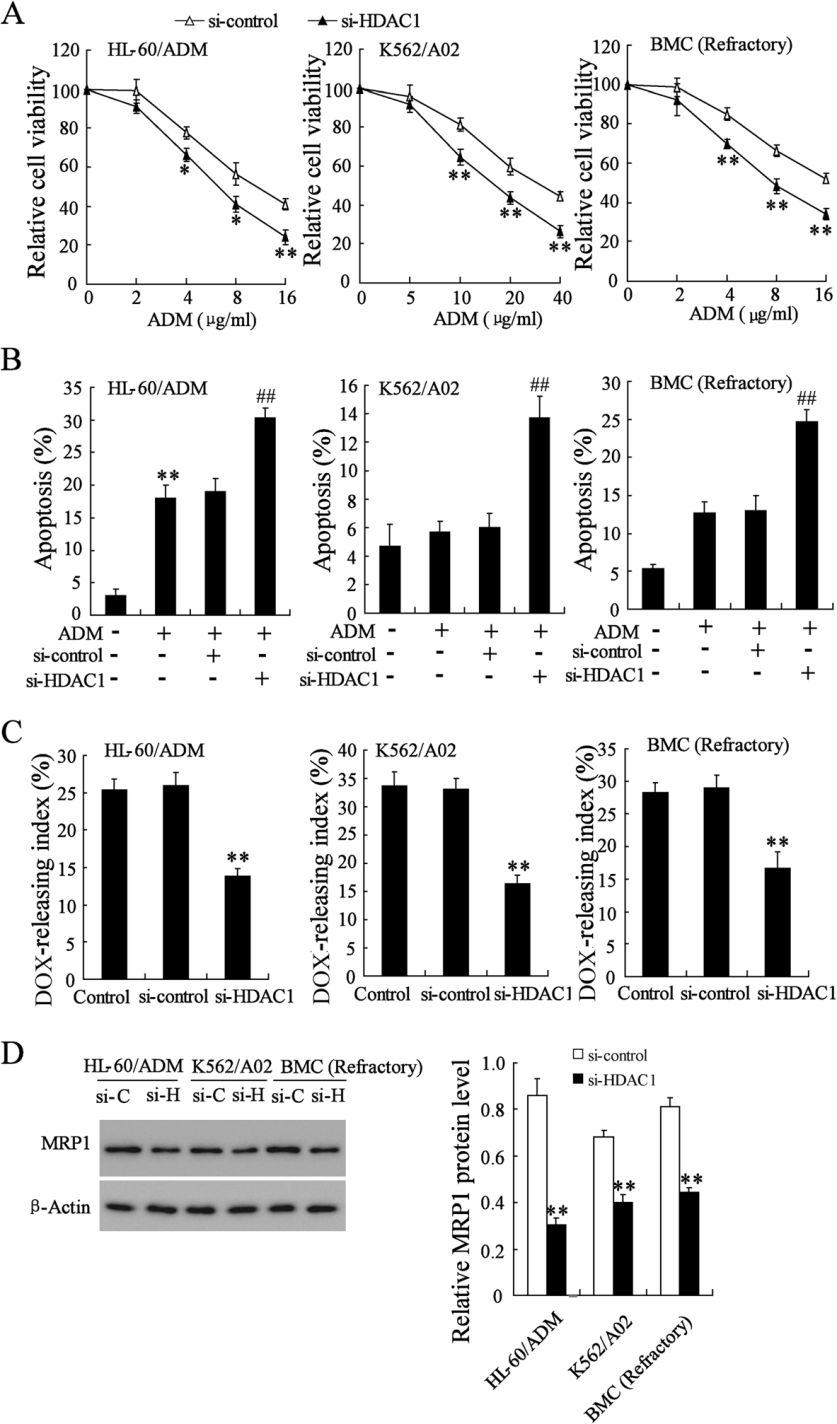
Effect of HDAC1 knockdown on doxorubicin resistance of HL-60/ADM, K562/A02, and primary BMCs of refractory AML. **a** Relative cell viability of si-HDAC1 transfected AML cells after different doses of doxorubicin treatment. **b** Percentage of apoptotic cells in si-HDAC1 transfected AML cells after doxorubicin (0.01 μg/ml) treatment. **c** doxorubicin-releasing index of si-HDAC1 transfected AML cells. **d** MRP1 protein expression in si-HDAC1 transfected AML cells. DOX, doxorubicin. Data are the mean ± SD of three independent experiments in AML cells. ^*^P < 0.05, ^**^P < 0.01 vs. si-control

Next, we knocked down HDAC1 via the specific RNAi sequence for HDAC1 in HL-60/ADM, K562/A02, and primary BMCs of refractory AML patients (Additional file 1: Figure S1C) and examined the effect of HDAC1 silencing on doxorubicin resistance. In contrast to the biological function of HDAC1 overexpression, HDAC1 knockdown remarkably improved the doxorubicin-induced inhibition on multidrug-resistant AML cells as shown by decreased cell viability and increased apoptotic cells (Fig. 3a, b). Moreover, HDAC1 silencing reduced the doxorubicin-releasing index of multidrug-resistant AML cells, which was positively correlated with cell multidrug-resistant (Fig. 3c). Consequently, MRP1 was also downregulated by HDAC1 knockdown (Figs. 3d). It was obvious that HDAC1 silencing may play a critical role in the therapeutic effect of doxorubicin on multidrug-resistant AML.

### HDACs inhibition decreased doxorubicin resistance of multidrug-resistant AML cells

**Fig. 4:**
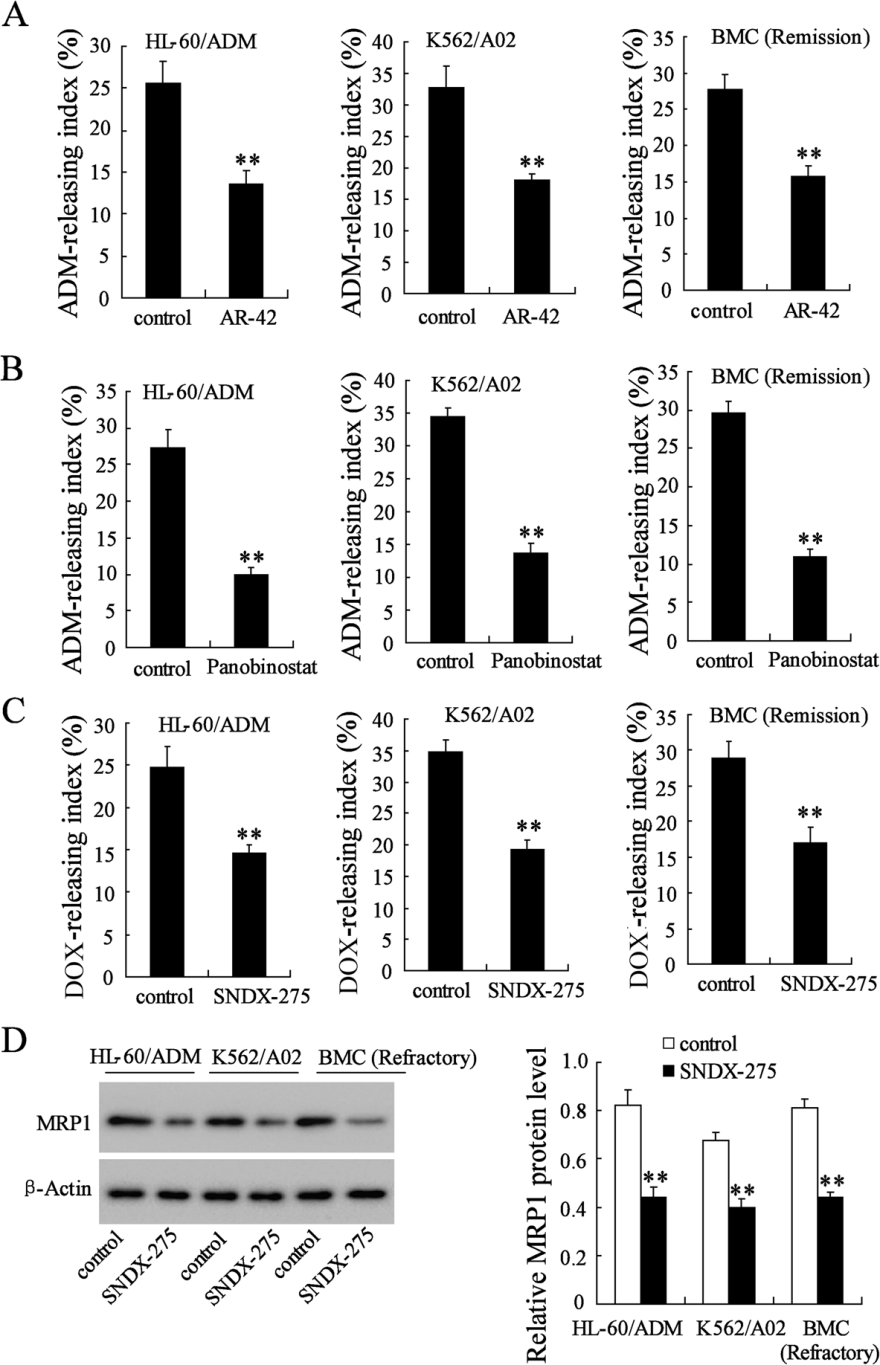
HDAC inhibition decreased doxorubicin resistance of HL-60/ADM, K562/A02 and primary BMCs in refractory AML. **a** doxorubicin-releasing index of AML cells after treatment with AR-42, an inhibitor of HDACs. **b** doxorubicin-releasing index of AML cells after treatment with Panobinostat, another inhibitor of HDACs. **c** doxorubicin-releasing index of AML cells after treatment with SNDH-275, a specific inhibitor of HDAC1. **d** MRP1 protein expression was detected by western blot. Data are the mean ± SD of three independent experiments in AML cells. ^**^P < 0.01 vs. control

To further determine the pro-drug resistance effect of HDACs, we performed flow cytometry analysis of the doxorubicin-releasing index in drug-resistant AML cells (HL-60/ADM, K562/A02, and primary BMCs of refractory AML) treated with an HDAC inhibitor. As shown in Fig. 4a, the doxorubicin-releasing index of cells incubated with the HDAC inhibitor AR-42 was significantly reduced. The doxorubicin-releasing indexes of cells incubated with the HDAC inhibitor Panobinostat or with HDAC1 specific inhibitor SNDX-275 were also significantly reduced (Fig. 4b, c). After SNDX-175 treatment, cellular protein level of MRP1 of these drug-resistant AML cells was attenuated (Fig. 4d). AR-42, Panobinostat and SNDX-275 promoted cell apoptosis of HL-60/ADM, K562/A02, and primary BMCs of refractory AML (Additional file 2: Figure S2). Taken together, these data suggested that inhibition of HDACs reduced doxorubicin resistance of drug-resistant AML cells.

### The high expression of HDAC1 protein was induced by post-translational regulation in multidrug-resistant AML cells

**Fig. 5:**
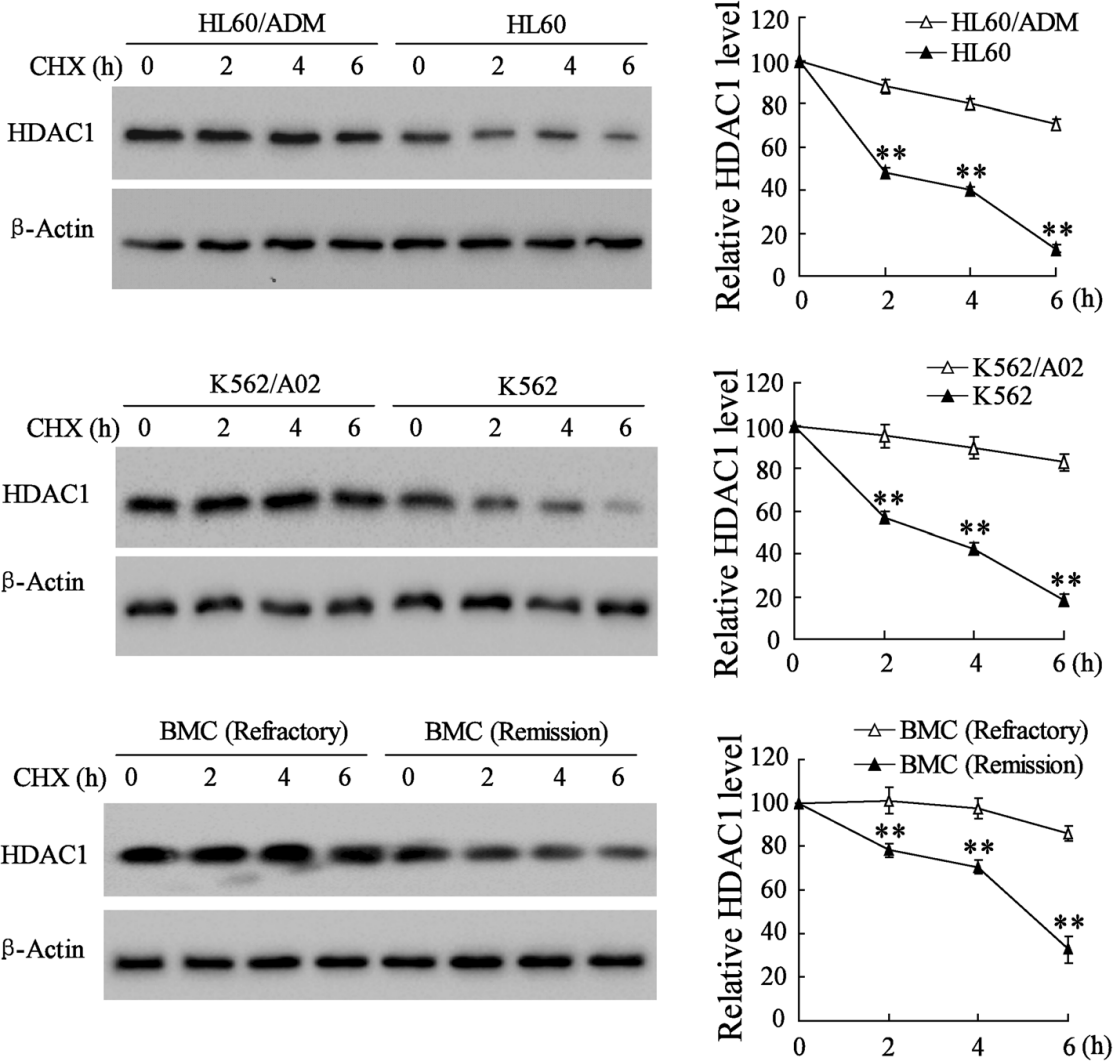
Western blot analyses of HDAC1 expression in HL-60, HL-60/ADM, K562, K562/A02, and BMCs of refractory AML and remission AML 2, 4, and 6 h after CHX treatment. Data are the mean ± SD of three independent experiments in AML cells. ^**^P < 0.01 vs. HL-60/ADM or K562/A02 or refractory BMCs

The control of the cellular protein level is a dynamic process involving protein synthesis and degradation. To detect the potential mechanism underlying the higher level of HDAC1 in drug-resistant AML cells, we first compared the differential expression of HDAC1 in nondrug-resistant AML cells and drug-resistant AML cells incubated with cycloheximide (CHX), a protein synthesis inhibitor (Fig. 5). With the time-course of CHX treatment, the expression of HDAC1 in HL-60, K562, and BMCs in remission AML was gradually downregulated in comparison to that of HL-60/ADM and K562/A02 cells in refractory AML. After 6 h of CHX treatment, the expression of HDAC1 showed the lowest level in HL-60 and K562 cells. These results suggested that the degradation of HDAC1 protein was, to some extent, inhibited in drug-resistant AML cells.

### HDAC1 underwent neddylation inhibition in AML

**Fig. 6:**
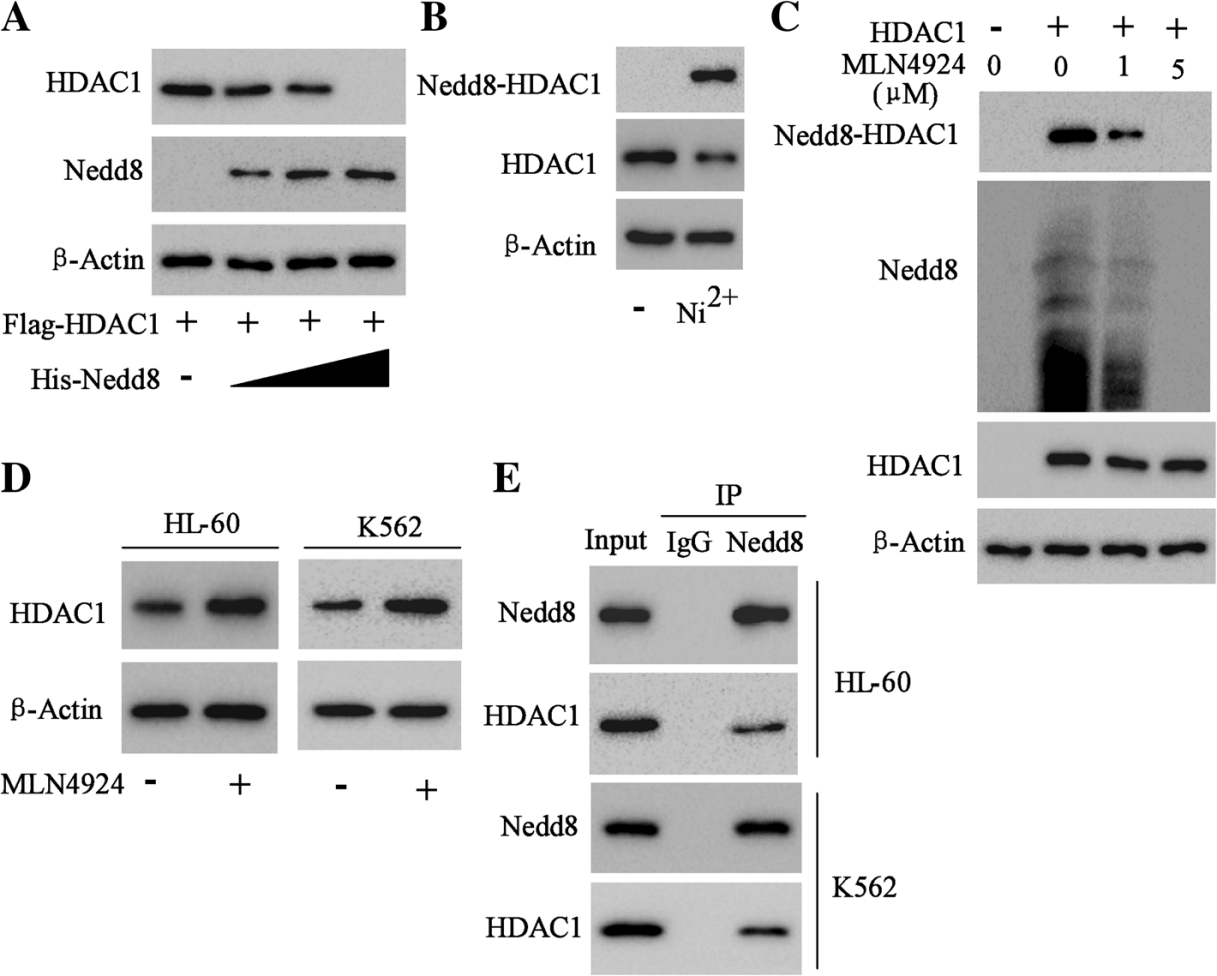
Neddylation of HDAC1. **a** Western blot analysis of Nedd8 substrates isolated by His purification in HL-60 cells co-transfected with Flag-HDAC1 and different dose of His-Nedd8. **b** Western blot analysis of Nedd8-HDAC1 (65kD) in the proteins purified by NTA-Ni^2+^ agarose beads from the HL-60 cells stably expressing His-Nedd8 by using primary antibody for HDAC1. HDAC1 (65kD) in HL-60 cells without His-Nedd8 transfection was also analyzed. **c** Western blot analysis of Nedd8 substrates of HL-60 cells expressing Flag-HDAC1 and His-Nedd8 after 16 h of treatment with MLN4924, an inhibitor of Nedd8-activating enzyme. **d** Western blot analysis of HDAC1 expression in HL-60 and K562 cells after MLN4924 treatment. **e** Immunoprecipitation assay of the interaction of HDAC1 and Nedd8 in HL-60 and K562 cells

We next sought to determine the mechanism in the degradation inhibition of HDAC1 protein in drug-resistant AML cells. Post-translational modifications were closely related to the target protein expression and function. HL-60 cells were co-transfected with Flag-HDAC1 and His-Nedd8 plasmids in different doses. The results showed that the level of HDAC1 protein decreased with Nedd8 increase in vitro (Fig. 6a). HL-60 cells were transfected with His-Nedd8 plasmid, and His-Nedd8-combined protein complexes were purified with nickel-agarose beads. As shown in Fig. 6b, HDAC1 was modified with the Nedd8-mediated neddylation in AML cells. Furthermore, MLN4924, a small molecule inhibitor of Nedd8-activating enzyme, could obviously inhibit the neddylation of HDAC1 (Fig. 6c). On the other hand, MLN4924 markedly elevated the protein level of HDAC1 both in HL-60 and K562 cells, suggesting that the high level of HDAC1 protein might be induced by the neddylation inhibition in AML cells (Fig. 6d). The assays of immunoprecipitation of HDAC1 and Nedd8 further confirmed the interaction between HDAC1 and Nedd8 in AML cells (Fig. 6e).

### Nedd8 facilitated the ubiquitination of HDAC1 in AML

**Fig. 7:**
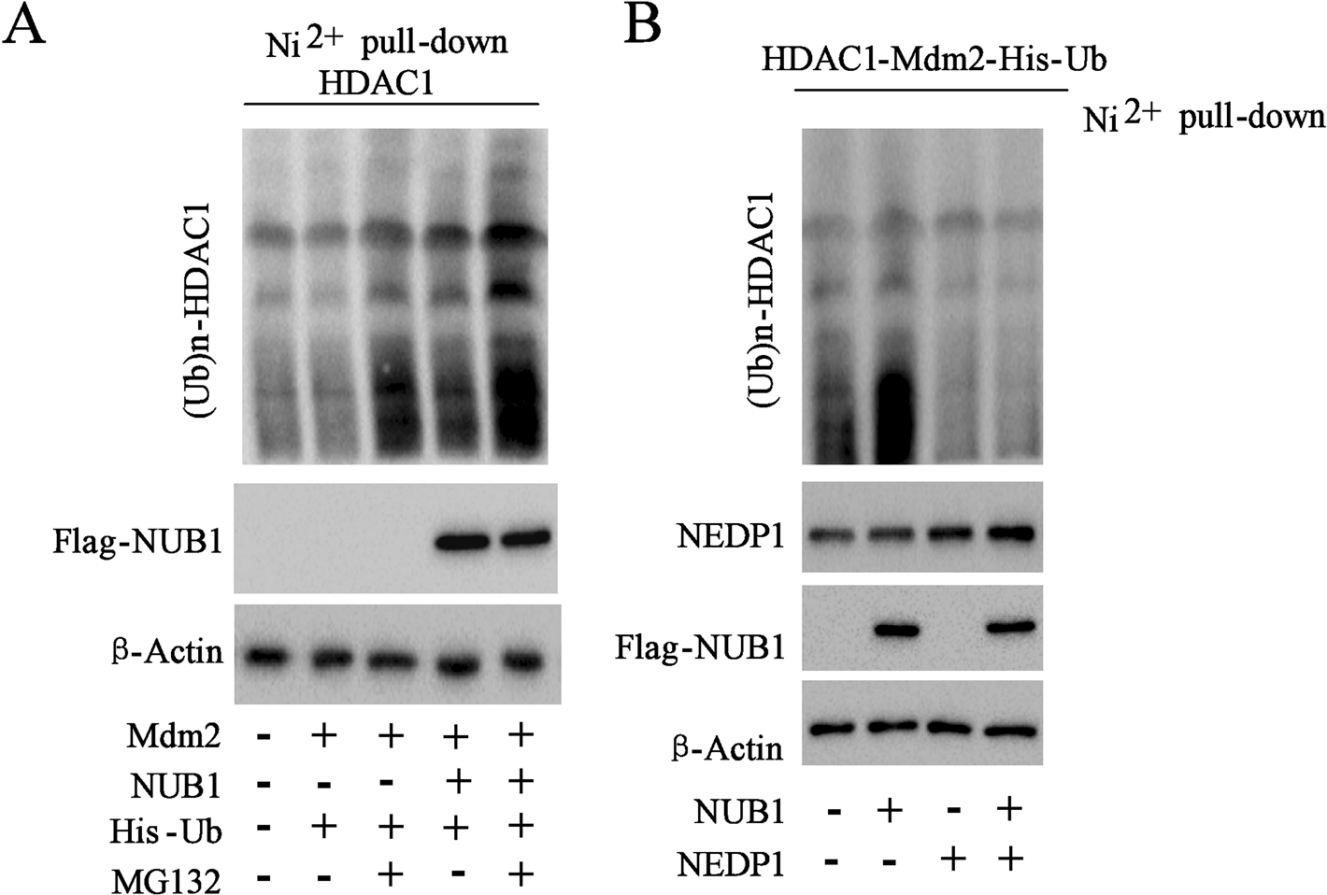
Nedd8 facilitated ubiquitination of HDAC1. **a** HL-60 cells transfected with constructs encoding HDAC1, His-ubiquitin (His-Ub), Flag-NUB1 (NEDD8 ultimate buster 1), and Myc-Mdm2 following incubation with MG132, a protease inhibitor. Ni2^+^ pull-down assay and Western blot analysis of (Ub)n-HDAC1 and Flag-NUB1 expression. **b** The Ni2^+^ pull down assay was evaluated by Western blot with appropriate antibodies in HL-60 cells expressing HDAC1, Flag-NUB1, His-ubiquitin, Myc-Mdm2, and NEDP1

Mdm-2, an E3 ligase, is well known to control the ubiquitination of HDAC1 [19]. Mdm-2 has dual specificity for ubiquitin and Nedd8 [21]. NUB1 (Nedd8 Ultimate Buster 1), a binding partner of Nedd8, was shown to cooperate with Nedd8 and ubiquitin to protein [22]. HL-60 cells were transfected with His-ubiquitin (His-Ub), Flag-NUB1 and Myc-Mdm2 as indicated. The data showed that Myc-Mdm2 expression in HL-60 cells did not affect the expression of (Ub)n-HDAC1, while NUB1 obviously facilitated the ubiquitination of HDAC1, which was further increased by the addition of proteasome inhibitor MG132 (Fig. 7a). Notably, NEDP1 with the capability of removing Nedd8 from its substrates [23], could suppress the ubiquitination of HDAC1 significantly in vitro (Fig. 7b). HDAC1 ubiquination of both the sensitive cell lines (HL-60 and K562) and resistant cell lines (HL-60/ADM and K562/A02) were tested and the results showed that ubiquination of the sensitive cell lines were stronger than those of resistant cell lines (Additional file 5: Figure S5). The results indicated that the ubiquitination of HDAC1 was also induced by Nedd8 in AML.

### HDAC1 overexpression promoted tumor growth in presence of doxorubicin in vivo tumor transplantation models

**Fig. 8:**
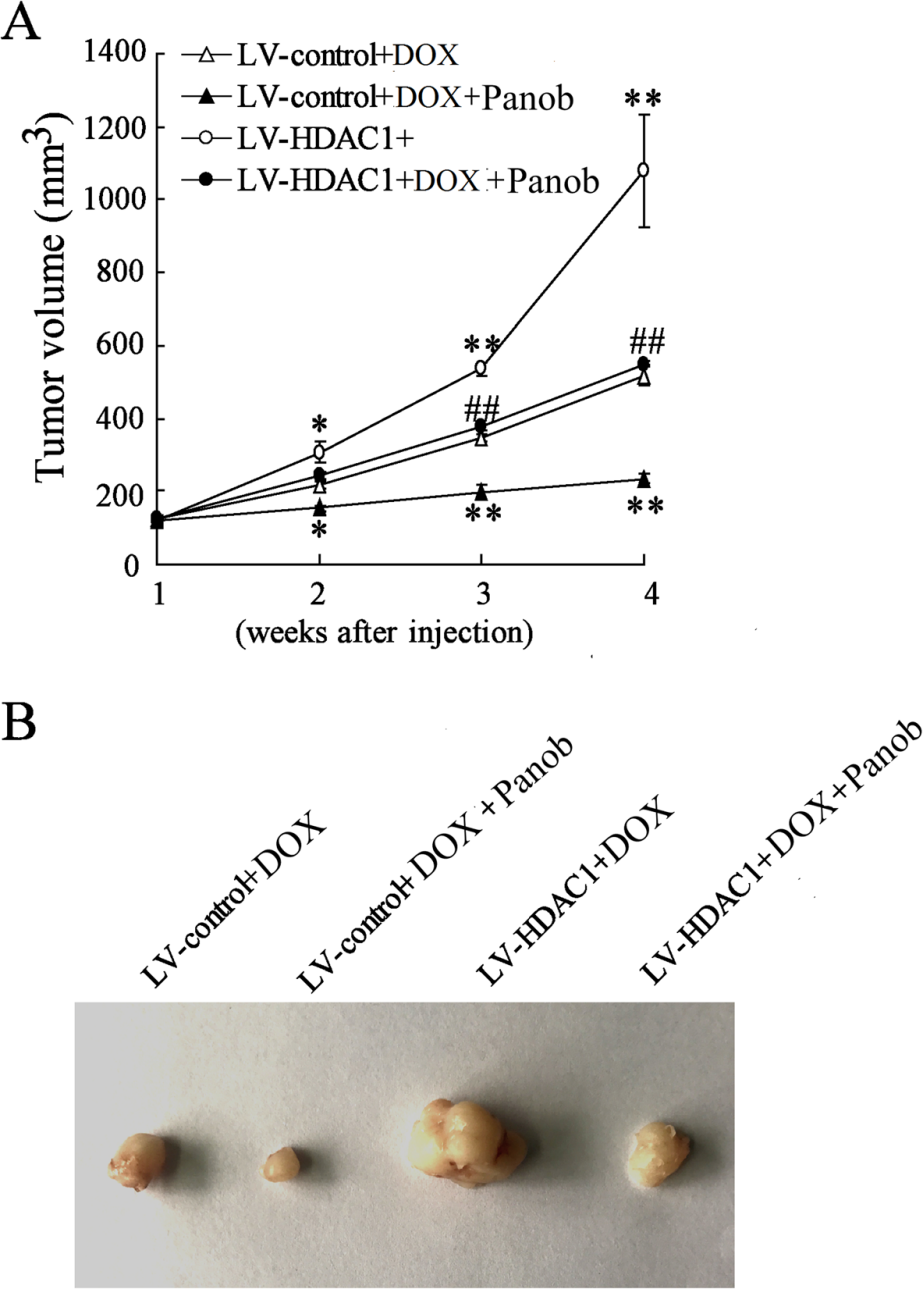
Tumor growth of HL-60 xenograft in mice. **a** Tumor volume of the HL-60 xenograft overexpressing HDAC1 in mice treated with doxorubicin or Panobinostat. **b** Representative tumor morphology. Panob, Panobinostat. DOX, doxorubicin. Data are the mean ± SD of six xenografts of mice. ^*^P < 0.05, ^**^P < 0.01 vs. LV control + doxorubicin, ^##^*P* < 0.01 vs. LV-HDAC1 + doxorubicin

Based on the findings pointing to the involvement of HDAC1 in multidrug resistance of AML cells, an AML cell xenograft mouse model was established and used to evaluate the effect of HDAC1 on the tumor growth in the presence of doxorubicin and Panobinostat treatment. HDACs inhibited by Panobinostat remarkably improved the inhibitory effect of doxorubicin on tumor growth in vivo, including tumor growth rates and volumes, while HDAC1 overexpression significantly increased doxorubicin resistance (Fig. 8a, b). Besides, HDACs inhibitor Panobinostat also greatly relieved the doxorubicin resistance induced by HDAC1 overexpression (Fig. 8a, b). The morphologies and sizes of the tumors induced by HL-60 cells were shown in Fig. 8b.

### HDAC1 knockdown inhibited tumor growth in presence of doxorubicin in vivo tumor transplantation models

**Fig. 9:**
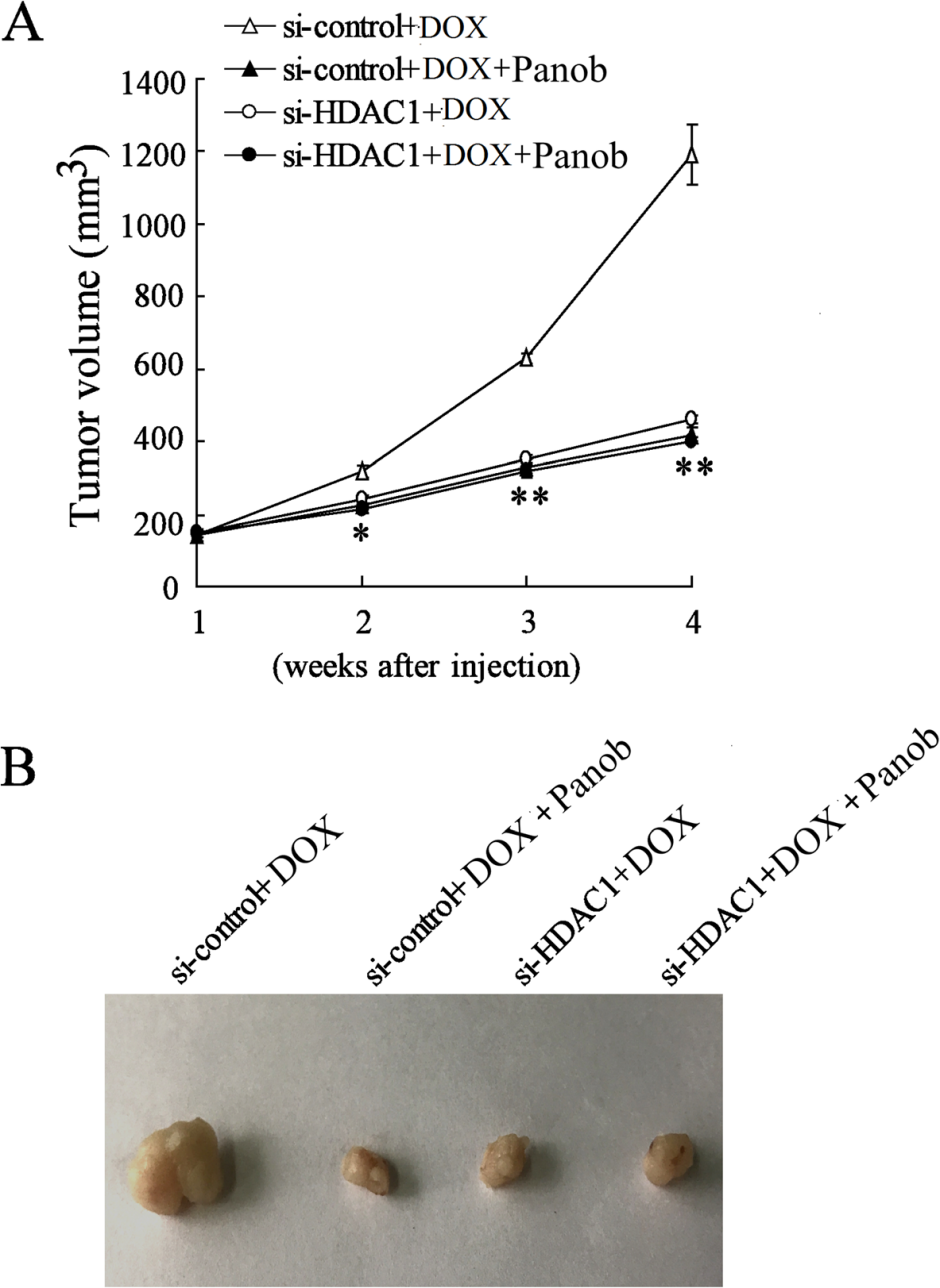
Tumor growth of HL-60/ADM xenograft in mice. **a** Tumor volume of the HL-60/ADM xenograft with HDAC1 silencing in mice treated with doxorubicin or Panobinostat. **b** Representative tumor morphology. Data are the mean ± SD of six xenografts of mice. ^*^P < 0.05, ^**^P < 0.01 vs. si-control + doxorubicin

For further definition of the role of HDAC1 in doxorubicin resistance, the models of AML bearing mice were induced by HL-60 cells and the lentiviral vector carrying interference sequence for HDAC1 was used to inhibit the expression of HDAC1 in vivo. As expected, HDAC1 silencing by Panobinostat and/or lentivirus mediated RNA interference against HDAC1 effectively reduced doxorubicin resistance, resulting in the inhibition of tumor growth in AML bearing mice (Fig. 9a, b).

## Discussion

HDACs are functional proteins, which are commonly recruited by gene promoters and regulate the acetylation balance of oncogenic or tumor suppressor fusion proteins. HDACs are indispensable epigenetic effectors, which play important roles in multidrug resistance [24]. At the post-translational regulation level, HDACs are known to be modified by ubiquitination and neddylation, which maintain the intracellular level [18, 19]. However, the potential involvement of HDAC expression and the regulatory roles of ubiquitination and neddylation of HDACs in AML multidrug resistance remain unclear. The present study highlighted the crucial role of HDAC1 in doxorubicin sensitivity of AML cells and identified the interplay of neddylation and ubiquitination in the regulation of intracellular HDAC1. The post-transcriptional regulation mechanism of neddylation of HDAC1 represents a novel therapeutic target for the induction of cell apoptosis in refractory AML.

Aberrant cellular epigenetic genes serve as key driver tumor onset and progression through transcriptional repression of tumor-suppressor genes on their promoters [25]. Also, post-translational alteration of HDAC was revealed in drug resistance [24]. Consequently, efforts have been made to develop HDACs inhibitors as anti-drug resistance agents for cancer therapeutics. For example, Ponnusamy L et al. found that HDAC inhibitor Trichostatin A significantly re-sensitized resistant breast cancer cells to doxorubicin [26]. To KKW and Fu LW found that HDAC and PI3K inhibitor reverses platinum drug resistance of cancer cells [27]. Kaowinn S et al. suggested that HDAC inhibition may sensitize cells to doxorubicin-induced apoptosis of lung cancer [28]. However, these studies did not point the specific HDAC isoform in drug resistance of cancer cell. In the present study, we comprehensively analyzed the potential involvement of HDAC patterns in drug resistance of AML. In particular, by determining HDAC patterns in multidrug-resistant AML patients and cells, as well as in nondrug-resistant AML patients and cells, we found that the upregulation of HDAC1 in multidrug resistance of AML cells. Combined with data showing that patients with relapsed/refractory AML had higher HDAC1 expression than newly diagnosed AML patients, we can infer that HDAC1 contributed to multidrug resistance of AML cells. Previously, studies have shown that the inhibition of HDAC1 regulated the apoptosis of tumor cells [29, 30], as well as that of AML cells [12, 31], by affecting its target genes. Based on the literature and the findings of the present study, we may suppose that HDAC1 is a progressive during resistance development of AML.

Subsequently, the present data provided strong evidence that overexpression of HDAC1 increased doxorubicin resistance of AML cells and that silencing of HDAC1 decreased doxorubicin resistance of AML cells by exerting effects on cell viability, cell apoptosis, the doxorubicin-releasing index, and MRP1 expression. Role of HDAC1 in our finding coincides with previous study in which HDAC1 silencing in ovarian cancer enhances the chemotherapy response [32]. HDAC has emerged as a potential therapeutic target, with inhibitors of HDAC used to treat AML in laboratory experiments and a clinical trial [33]. In the present study, two HDAC inhibitors, Panobinostat and AR-42, were used to confirm the involvement of HDAC1 in drug resistance of AML cells. Panobinostat has been widely evaluated in the treatment of AML cells and AML models [34, 35]. AR-42 has been used in a phase 1 trial for multiple myeloma and in a clinical trial for T- and B-cell lymphomas [36]. The findings of the present study suggested that both these inhibitors increased doxorubicin sensitivity of drug-resistant AML cells and primary BMCs in refractory AML by attenuating the doxorubicin-releasing index. Furthermore, doxorubicin sensitivity of drug-resistant AML cells and primary BMCs in refractory AML was also re-sensitized by the specific inhibitor of HDAC1, SNDX-275. Also SNDX-275 contributes to downregulation of MRP1, a drug resistance protein, in drug-resistance cell and primary BMCs of remission AML. These data suggest the positive role of HDAC1 in the drug resistance of AML cells. Through the detail mechanism by which HDAC1 promoting drug resistance of AML remains unclear, we found that in HL-60/ADM cells, K562/A02 cells and primary BMCs of refractory AML, HDAC1 downregulating Apoptotic protease activating factor 1 (Apaf-1), a putative tumor suppressor gene that has been reported to closely correlated with drug resistance in lymphoma [37, 38] and as well as in AML [39], through directly combined with its promoter (Additional file 3: Figure S3).

Mechanistic studies have determined that dysfunction of protein synthesis and protein degradation contributed to the phenotypic characteristics of AML [40], pointing to the importance of maintaining the intracellular protein balance. Catalysis by protein synthases is the first important step in protein synthesis. In the present study, treatment with the protein synthesis inhibitor CHX resulted in downregulation of HDAC1 in nondrug-resistant AML cells, but it had no effect on HDAC1 expression in drug-resistant AML cells. This finding indicated that the elevated expression level of HDAC1 in drug-resistant AML cells was not dependent on the protein synthesis process. Previous studies indicated that dysfunction of the degradation of drug resistant-associated proteins, mediated by neddylation or ubiquitination, might be the mechanism underlying multidrug resistance [41, 42]. The data in the present study suggested that HDAC1 was a substrate for Nedd8 in AML cells and that the addition of MLN4924, an inhibitor of Nedd8-activating enzyme [43], enhanced HDAC1 expression. Interestingly, we observed interplay between neddylation and ubiquitination of HDAC1 in AML cells via an Mdm2-dependent mechanism. Mdm2, an E3 ligase, which controls gene modification by ubiquitin, was recently identified as a dual specific molecule for ubiquitin and Nedd8 [21]. A previous study also identified the interplay between neddylation and ubiquitination [22]. However, the present study is the first to identify the combined effect of neddylation and ubiquitination on the regulation of HDAC1 expression in AML cells. Although HDAC1 also can be modified by sumoylation [44], we found that sumoylation of HDAC1 in AML cells did not affect the ubiquitination and degradation of HDAC1 (data not shown). One interesting finding in the present study was the identification of the positive role of HDAC1 in doxorubicin resistance of AML cells, which was confirmed by the AML cell xenograft mouse model. The HDAC expression pattern in drug-resistant AML has not been described previously. The present study is the first to identify the HDAC1 expression pattern in AML and identify HDAC1 as a positive regulator of multidrug resistance in AML cells.

## Conclusions

In summary, although details of the regulatory mechanism underlying intracellular HDAC1 control are unknown, the results of the present study indicated that post-translational modification, (i.e., neddylation and ubiquitination) may be involved in the regulation of HDAC1 expression in AML patients or AML cells. The observations of the positive impacts of siRNA HDAC1 and Panobinostat in the AML xenograft model point to the potential possibility of their use as combination therapy for refractory AML.

## Additional files

Additional file 1: **Figure S1.** Western blot analysis of (A) acetyle-histone 3 (Ac-H3) and (B) acetyle-histone 4 (Ac-H4) in remission AML and refractory AML, (C) HDAC1 in indicated cells after HDAC1 overexpression or interference, and (D) MRP1 in AML at the state of the newly diagnosed and relapsed/refractory. (TIF 2203 kb)

Additional file 2: **Figure S2.** Apoptosis of primary BMCs after treatment of AR-42, panobinostat and SNDX-275 in refractory patient was determined by flow cytometry method. (TIF 710 kb)

Additional file 3: **Figure S3.** HDAC1 affects Apaf-1 expression in HL-60/ADM, K562/A02 and BMCs (Refractory). (A) ChIP assay of combination of HDAC1 and Apaf-1 promoter. (B) QRT-PCR analysis of Apaf-1 expression in cells after si-HDAC1transfection. ^**^P < 0.01 vs. si-control. (TIF 459 kb)

Additional file 4: **Figure S4.** The growth inhibition rate of HL-60, K562, K562/A02 and HL-60/ADM cells. (TIF 335 kb)

Additional file 5: **Figure S5.** Ubiquination of HDAC1 in both the sensitive cell lines (HL-60 and K562) and resistant cell lines (HL-60/ADM and K562/A02). (TIF 537 kb)

## Data Availability

Not applicable

## Abbreviations

Ac-H3: Acetyle-histone 3
AML: Acute myelogenous leukemia
BMCs: Bone marrow cells
CHX: Cycloheximide
HDACs: Histone deacetylases
His-Ub: His-ubiquitin
ITDs: Internal tandem duplications
MRP1: Multidrug resistance associated protein 1
SD: Standard deviation
si-HDAC1: siRNA constructs silencing HDAC1
