# Supplementary figures and images for: Histone deacetylase 1 induced by neddylation inhibition contributes to drug resistance in acute myelogenous leukemia
Source: Cell Commun Signal. 2019 Jul 29;17:86. doi: 10.1186/s12964-019-0393-8 (PMC6664585; doi:10.1186/s12964-019-0393-8)

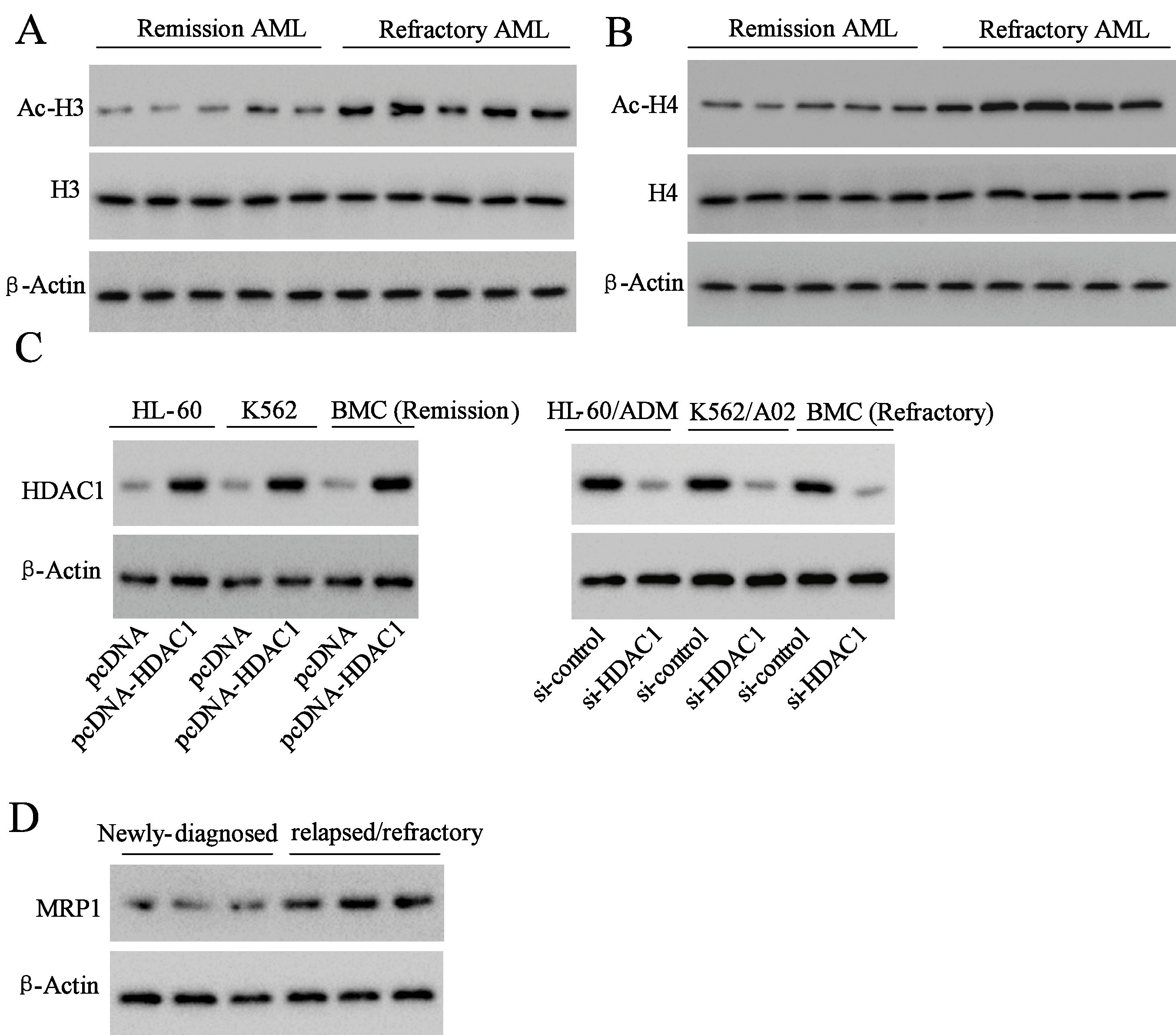

Supplement: Supplementary file 1 — Figure S1. Western blot analysis of (A) acetyle-histone 3 (Ac-H3) and (B) acetyle-histone 4 (Ac-H4) in remission AML and refractory AML, (C) HDAC1 in indicated cells after HDAC1 overexpression or interference, and (D) MRP1 in AML at the state of the newly diagnosed and relapsed/refractory. (TIF 2203 kb) [file 12964_2019_393_MOESM1_ESM.tif]

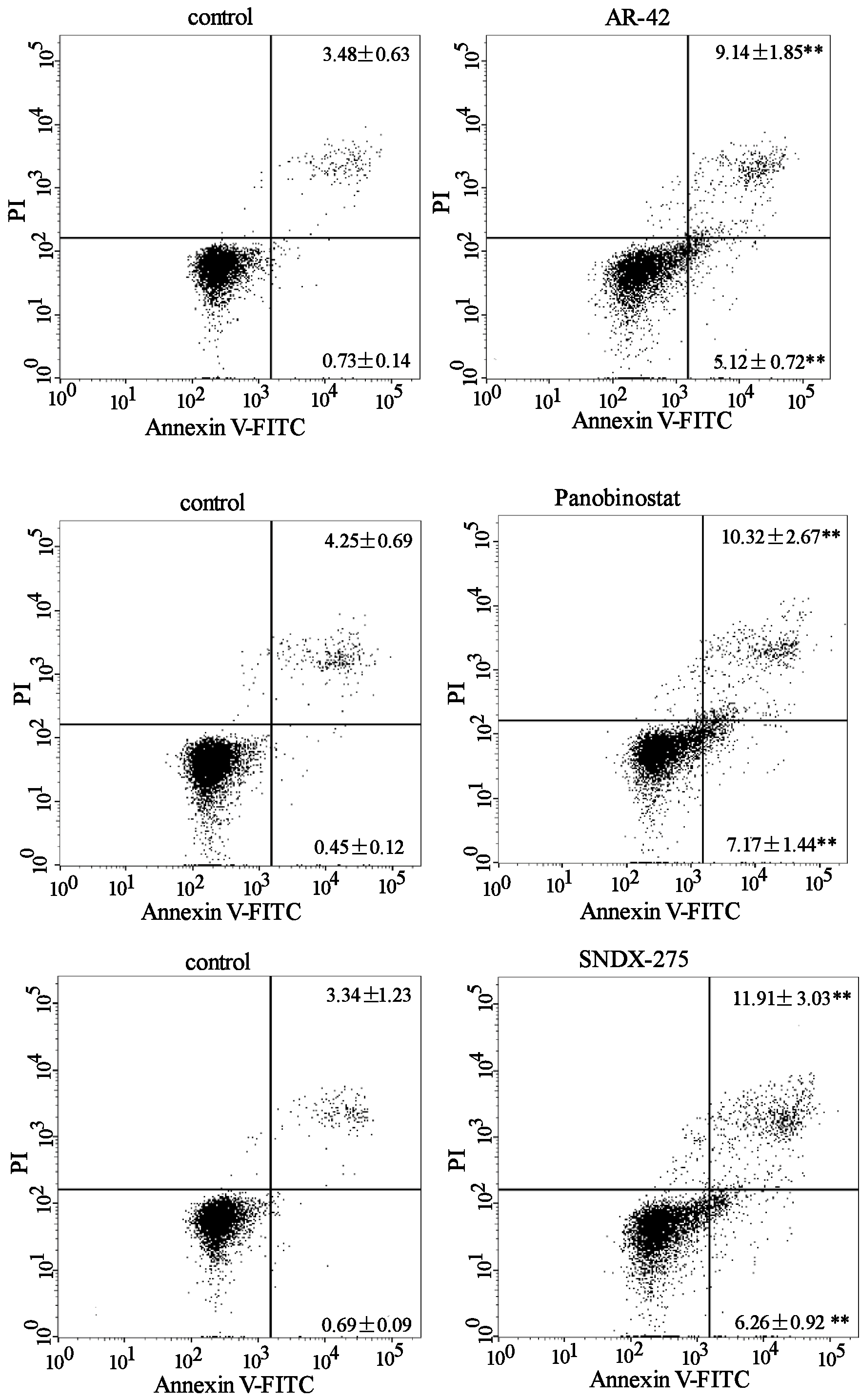

Supplement: Supplementary file 2 — Figure S2. Apoptosis of primary BMCs after treatment of AR-42, panobinostat and SNDX-275 in refractory patient was determined by flow cytometry method. (TIF 710 kb) [file 12964_2019_393_MOESM2_ESM.tif]

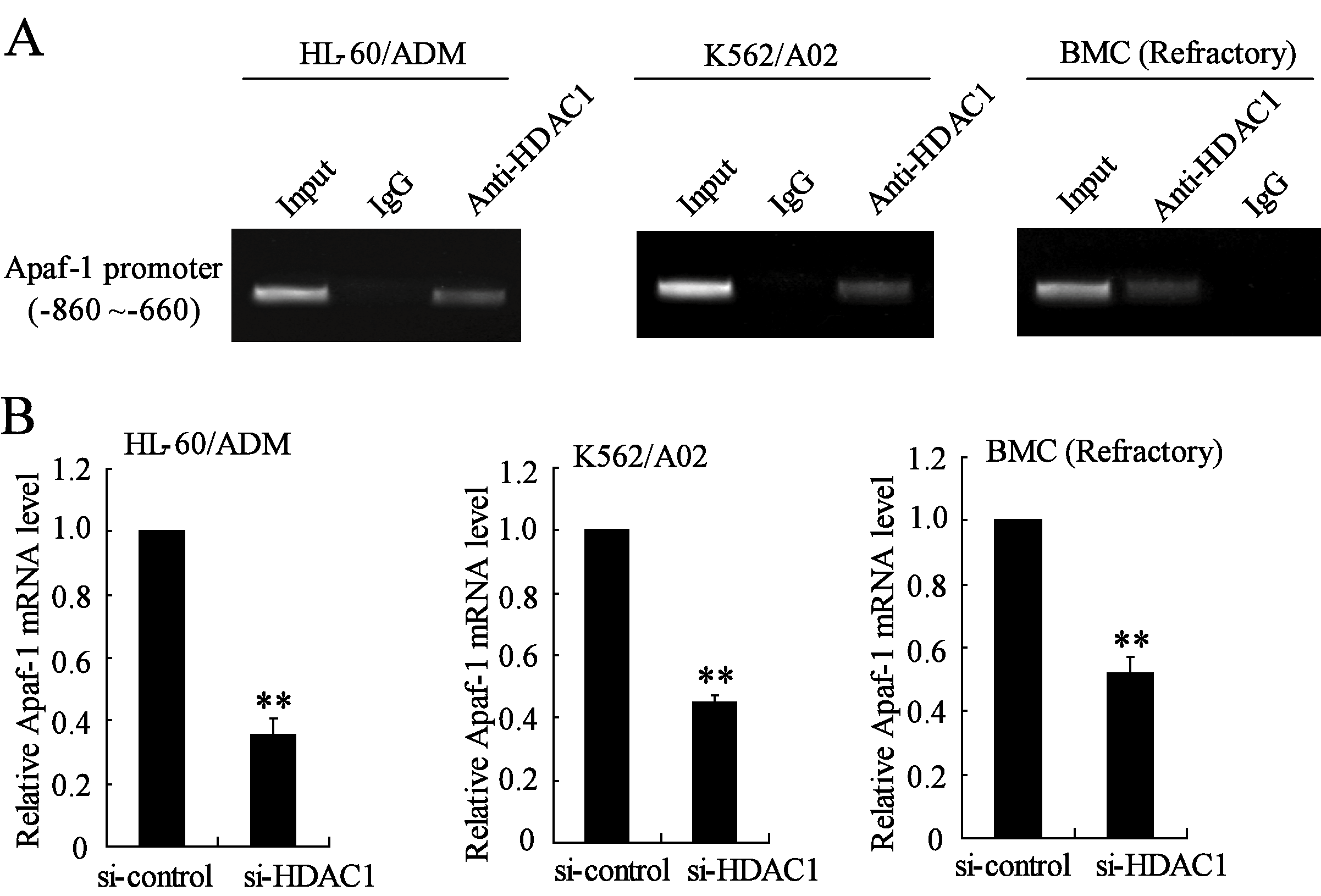

Supplement: Supplementary file 3 — Figure S3. HDAC1 affects Apaf-1 expression in HL-60/ADM, K562/A02 and BMCs (Refractory). (A) ChIP assay of combination of HDAC1 and Apaf-1 promoter. (B) QRT-PCR analysis of Apaf-1 expression in cells after si-HDAC1transfection. **P < 0.01 vs. si-control. (TIF 459 kb) [file 12964_2019_393_MOESM3_ESM.tif]

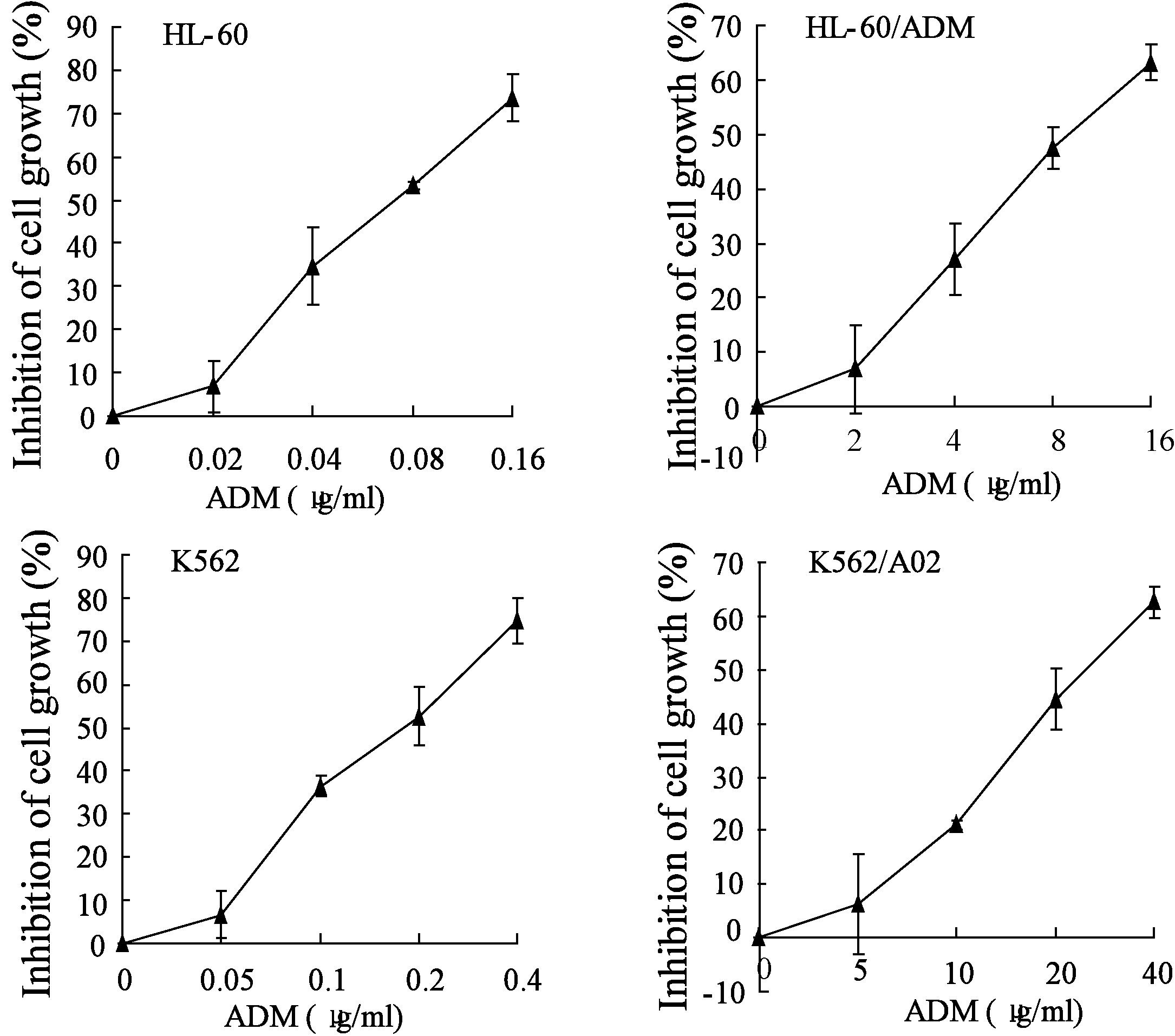

Supplement: Supplementary file 4 — Figure S4. The growth inhibition rate of HL-60, K562, K562/A02 and HL-60/ADM cells. (TIF 335 kb) [file 12964_2019_393_MOESM4_ESM.tif]

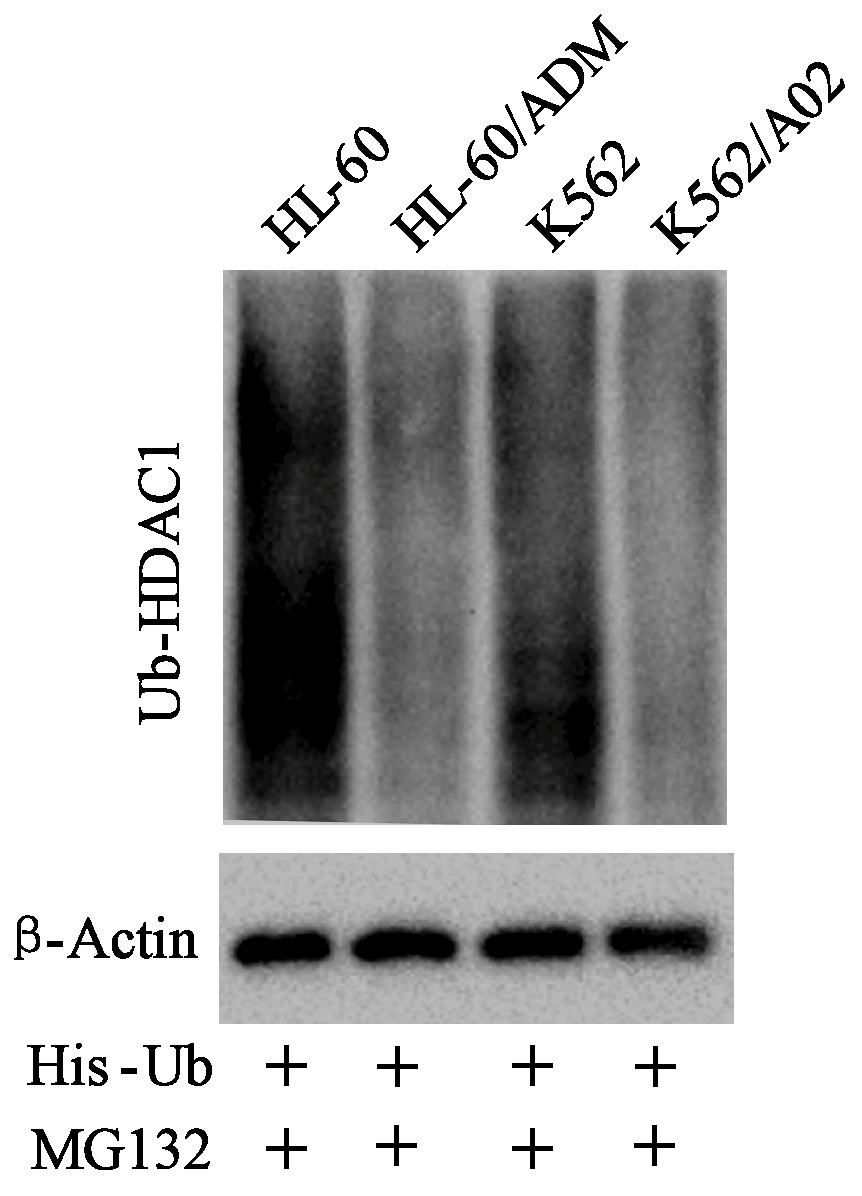

Supplement: Supplementary file 5 — Figure S5. Ubiquination of HDAC1 in both the sensitive cell lines (HL-60 and K562) and resistant cell lines (HL-60/ADM and K562/A02). (TIF 537 kb) [file 12964_2019_393_MOESM5_ESM.tif]
